# Changes in nationwide thyroid hormone replacement use during the pandemic: results from the Trends in Drug Utilization During COVID-19 Pandemic in Turkey (PANDUTI-TR) Study

**DOI:** 10.1007/s00210-025-04310-9

**Published:** 2025-06-06

**Authors:** Caner Vizdiklar, Volkan Aydin, Onur Gultekin, Gokhan Tazegul, Ahmet Akici

**Affiliations:** 1 Department of Medical Pharmacology, School of Medicine, Marmara University, Istanbul, Turkey; 2 Turkish Medicines and Medical Devices Agency, Ankara, Turkey; 3 Department of Basic Medical Sciences / Pharmacology, School of Dentistry, Marmara University, Istanbul, Turkey; 4 Division of General Internal Medicine, Department of Internal Medicine, School of Medicine, Marmara University, Istanbul, Turkey

**Keywords:** Hypothyroidism, Levothyroxine, COVID-19 restrictions, Drug utilization

## Abstract

The COVID-19 pandemic and associated measures have had significant impacts on healthcare services, including medication use. We aimed to investigate the nationwide changes in thyroid hormone preparation (THP) utilization before, during, and after the COVID-19 restrictions. We obtained nationwide outpatient THP sales and projected prescribing data for the period between 01.03.2018 and 31.12.2022 from IQVIA Turkey. The average monthly THP consumption, cost, and quarterly projected prescribing levels were analyzed across three periods: “before restrictions” (BfR, 01.03.2018–31.03.2020), “during restrictions” (DuR, 01.04.2020–31.03.2022), and “after restrictions” (AfR, 01.04.2022–31.12.2022). Drug consumption levels were calculated using DID (defined daily dose per 1000 inhabitants per day) parameter. Mean monthly consumption of THPs across BfR, DuR, and AfR was 15.8 ± 3.0, 18.2 ± 4.0 (*p* > 0.05), and 21.1 ± 3.7 DID (*p* = 0.001 vs. BfR), respectively. Cost of THPs increased from €2.5 m ± 0.4 m in BfR to €2.8 m ± 0.6 m in DuR (*p* > 0.05), and €3.5 m ± 0.7 m in AfR (*p* < 0.001 vs. BfR, *p* = 0.009 vs. DuR). Projected prescribing levels of these drugs declined from 6.9 ± 0.4 DID in BfR to 6.2 ± 0.5 DID in DuR (*p* = 0.005), then rose to 7.3 ± 0.03 DID in AfR (*p* = 0.003 vs. DuR). Ongoing THP users accounted for 89.4% of all projected THP prescriptions and followed the overall trend. Projected prescribing for new users remained stable across three periods (*p* > 0.05). This study demonstrated that THP utilization showed a modest upward trend since the onset of the COVID-19 pandemic. The increase in consumption following the relaxation of restrictions might be associated with a potentially elevated need for pharmacotherapy, likely due to reduced healthcare access during the extraordinary conditions.

## Introduction

The extraordinary circumstances brought on by the coronavirus disease 2019 (COVID-19) pandemic have presented significant challenges regarding the diagnosis, treatment, and ongoing follow-ups of individuals with acute and chronic medical conditions (Boelaert et al. 2020). In response, international and national authorities have deployed a range of management strategies to address the issues in accessing healthcare services and medications (Núñez et al. 2021; Miller et al. 2021). Furthermore, COVID-19 has been reported to induce various subclinical manifestations across multiple organ systems, even in mild-to-moderate cases (Petersen et al. 2022). The thyroid gland expresses angiotensin-converting enzyme 2 (ACE2), the primary receptor facilitating SARS-CoV-2 entry to host cells (Lazartigues et al. 2020). Indeed, recent research has indicated that thyroid dysfunction might arise both during and post COVID-19 infection (Rossetti et al. 2022).

Management of hypothyroidism involves regular measurements of plasma thyroid hormone parameters and very slight dose adjustments in thyroid hormone replacement therapy, within the limits of targeted hormone levels (Surana et al. 2017; Hennessey and Espaillat 2018). Adherence to treatment was shown to be at moderate levels, and factors such as regular healthcare visits and adequate knowledge about the treatment reportedly contribute to higher adherence (Kumar and Shaukat 2019). Considering these characteristics, it has been suggested that patients with hypothyroidism could be affected by the pandemic-induced limitations on healthcare access and associated negative outcomes (Boelaert et al. 2020). Consequently, thyroid working groups have issued specific recommendations for managing these patients, including maintaining pre-pandemic doses and prescribing medications for euthyroid patients in intervals of 3 to 4 months to reduce the number of visits during that period (Pramono 2020; Rossetti et al. 2022). All these factors might have influenced drug consumption and prescribing patterns. Moreover, the profound economic impact of the pandemic led to increased financial vulnerability (Açıkgöz and Günay 2021), which has been reported to contribute to cost-related declines in medication adherence (Ruksakulpiwat et al. 2022)—an outcome that may likewise be reflected in altered patterns of thyroid hormone preparation (THP) use and the associated drug expenditures. This underscores the need for comprehensive studies demonstrating the influence of the COVID-19 pandemic on the disease burden of hypothyroidism through the changing pharmacotherapy trends. In this context, this study aimed to investigate whether the consumption, prescribing, and costs of medications used in hypothyroidism treatment were impacted by national drug access practices specific to the pandemic period.

## Methods

### Study design

This study was the hypothyroidism treatment-related section of the comprehensive “Trends in Drug Utilization During COVID-19 Pandemic in Turkey” (PANDUTI-TR) project, which evaluated drug consumption, prescribing, and expenditure data for a total of six metabolic and psychiatric indications (Vizdiklar et al. 2024). The changes in the consumption, prescribing, and costs of THPs across Turkey were examined in relation to alterations in COVID-19 pandemic restrictions. The study was approved by Marmara University School of Medicine Ethics Committee for Clinical Studies (approval number: 09.2022.825).

### Data collection

The data used in the study were sourced from IQVIA Turkey, which provides national-scale pharmaceutical market sales data across various countries (Kirmizi et al. 2022). Data on the wholesale distribution of drugs from pharmaceutical warehouses to community pharmacies across Turkey were used for drug consumption and expenditure analyses. This consumption dataset encompasses all healthcare sectors and represents approximately 91.0% of total retail pharmaceutical market sales throughout the years spanning the study period (IQVIA Turkey 2024). The drugs included in the study were selected based on the Anatomical Therapeutic Chemical (ATC) classification system, specifically those classified under “H03AA-thyroid hormones” at the fourth level (WHO Collaborating Centre for Drug Statistics Methodology 2024). Accordingly, the monthly number of units (i.e., packs) sold and their costs for THPs from March 1, 2018 to December 31, 2022 were obtained. Prescribing trends of THPs issued for hypothyroidism (ICD-10 codes: E01, E02, E03) were examined using quarterly projected nationwide prescribing data covering the corresponding timeframe. This dataset was compiled using outpatient prescriptions collected from 1000 physicians over a 7-day span to estimate the nationwide number of units prescribed per quarter (Vizdiklar et al. 2024). Similar to the consumption data, it covers all healthcare sectors.

### Measures

The mean monthly THP consumption levels and costs were calculated and compared across three distinct periods: “before restrictions” (BfR, from March 1, 2018 to March 31, 2020), “during restrictions” (DuR, from April 1, 2020 to March 31, 2022), and “after restrictions” (AfR, from April 1, 2022 to December 31, 2022). These time periods were established according to the dates when COVID-19 pandemic-related restrictions were enacted and lifted in Turkey, also considering potential delays in the impact of these restrictions on retail-level drug consumption (Demirbilek et al. 2020; Turkish Ministry of Interior 2022). Drug consumption was quantified in units and defined daily doses (DDD)/1000 inhabitants/day (DID), where applicable. DID is a metric established for the measurement of drug consumption across a population in a specific area, and based on DDD of a drug, i.e., the average maintenance dose per day for its primary use in adults (WHO Collaborating Centre for Drug Statistics Methodology 2024). Expenditure stemming from drug consumption was calculated and compared based on average monthly costs across BfR, DuR, and AfR. Cost values, initially obtained in Turkish liras, were converted to euros using the euro/Turkish lira exchange rate periodically declared by the Turkish Ministry of Health, which was 2.6934 at the beginning and 10.7577 at the end of the study period (Turkish Medicines and Medical Devices Agency 2018, 2022). Mean quarterly projected prescribing levels of THPs, expressed in units and DID, were assessed and compared across BfR, DuR, and AfR, with results stratified by new and ongoing users. Furthermore, the changes in consumption and projected prescribing of levothyroxine-containing preparations in units, categorized by strength, were also evaluated and compared.

### Statistical analysis

Statistical analyses were performed using GraphPad Prism 10.1 and IBM SPSS 29.0 software. Results were presented as numbers and percentages or means ± standard deviation, where appropriate. Shapiro–Wilk test was used to examine the normality of distribution for continuous variables. One-way analysis of variance (ANOVA) test with Tukey’s post-hoc test was used to compare normally distributed data, while Kruskal–Wallis test with Dunn’s post-hoc test was conducted in cases of non-normal distribution. Statistical significance was inferred for type 1 error values less than 5%.

## Results

The total amount and cost of THPs consumed throughout the 58-month study period were 103.4 million units and €162.2 m, respectively. The projected data of prescriptions with a hypothyroidism diagnosis included a total of 37.7 million units of THPs. Of the THPs consumed, 99.9% contained the active ingredient levothyroxine, while 0.1% contained liothyronine.

**Fig. 1:**
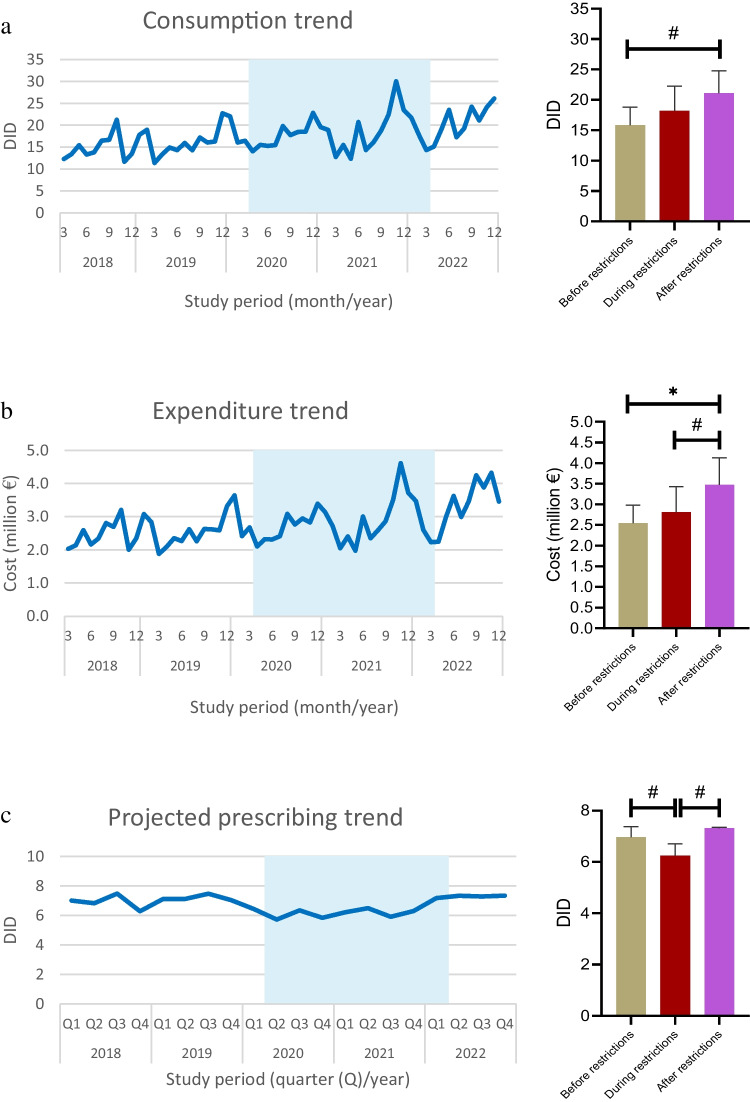
Trends and comparisons of average **a** monthly consumption, **b** monthly expenditure, and **c** quarterly projected prescribing of thyroid hormone preparations across COVID-19 restriction–associated periods. *p < 0.001; #*p* < 0.01

Mean monthly consumption (i.e., number of THP units distributed from pharmaceutical wholesalers to community pharmacies) levels of THPs were 15.8 ± 3.0 DID in BfR, 18.2 ± 4.0 DID in DuR (*p* > 0.05), and 21.1 ± 3.7 DID in AfR (*p* = 0.001 vs. BfR), (Table 1, Fig. 1a). Average monthly cost of THPs reached from €2.5 ± 0.4 m in BfR to €2.8 ± 0.6 m in DuR (*p* > 0.05), and later to €3.5 ± 0.7 m in AfR (*p* < 0.001 vs. BfR, *p* = 0.009 vs. DuR) (Table 1, Fig. 1b).

**Table 1:** Comparison of mean monthly consumption, monthly expenditure and quarterly projected prescribing levels of thyroid hormone preparations across COVID-19 pandemic-related restriction periods

Thyroid hormone preparations		Before restrictions (mean ± SD)	During restrictions (mean ± SD)	After restrictions (mean ± SD)	Overall *p*-value	Post-hoc *p*-value^*^
Consumption	Units	1,639,775.6 ± 285,742.2	1,835,612.6± 408,956.3	2,041,059.0 ± 348,562.6	0.012	0.013 (BfR vs. AfR)
	DID	15.8 ± 3.0	18.2±4.1	21.1 ± 3.7	0.001	0.001 (BfR vs. AfR)
Expenditure (€)		2,544,064.6 ± 435,461.8	2,808,408.3 ± 619,626.2	3,469,298.6 ± 659,647.7	< 0.001	< 0.001 (BfR vs. AfR) 0.009 (DuR vs. AfR)
Projected prescribing	Units	1,832,083.4 ± 102,766.6	1,814,334.5± 185,031.2	2,222,896.1 ± 49,278.0	0.001	0.002 (BfR vs. AfR) 0.001 (DuR vs. AfR)
	DID	6.9 ± 0.4	6.2 ± 0.5	7.3 ± 0.03	0.001	0.005 (BfR vs. DuR) 0.003 (DuR vs. AfR)
	*For ongoing users*	6.2 ± 0.4	5.5 ± 0.4	6.5 ± 0.04	0.002	0.015 (BfR vs. DuR) 0.004 (DuR vs. AfR)
	*For new users*	0.7 ± 0.1	0.7 ± 0.1	0.8 ± 0.05	0.487	-

Consumption refers to the number of thyroid hormone preparation units distributed from pharmaceutical wholesalers to community pharmacies. Projected prescribing refers to the estimated number of thyroid hormone preparation units prescribed for hypothyroidism. * Only statistically significant post-hoc *p*-values were reported

**Fig. 2:**
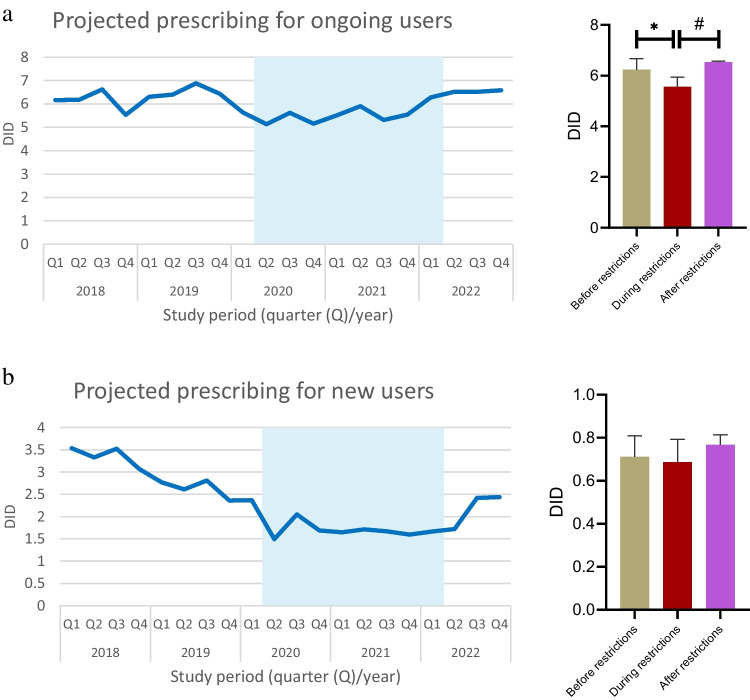
Trends and comparisons of average quarterly projected prescribing of thyroid hormone preparations for **a** ongoing and **b** new users across COVID-19 restriction–associated periods. *p = 0.015; #*p* = 0.004

The average quarterly projected prescribing (i.e., estimated number of THP units prescribed for hypothyroidism) levels of THPs regressed from 6.9 ± 0.4 DID in BfR to 6.2 ± 0.5 DID in DuR (*p* = 0.005), only to increase back to 7.3 ± 0.03 DID in AfR (*p* = 0.003 vs. DuR, Table 1, Fig. 1c). Of all THPs prescribed, 89.4% were issued to ongoing users, and 10.6% to first-time users. Projected THP prescribing levels for ongoing users declined from 6.2 ± 0.4 DID in BfR to 5.5 ± 0.4 DID in DuR (*p* = 0.015), then elevated to 6.5 ± 0.04 DID in AfR (*p* = 0.004 vs. DuR) (Table 1, Fig. 2a). Projected prescribing levels for new users remained stable, with 0.7 ± 0.1 DID in BfR, 0.7 ± 0.1 DID in DuR, and 0.8 ± 0.05 DID in AfR (*p* > 0.05, Table 1, Fig. 2b).

**Fig. 3:**
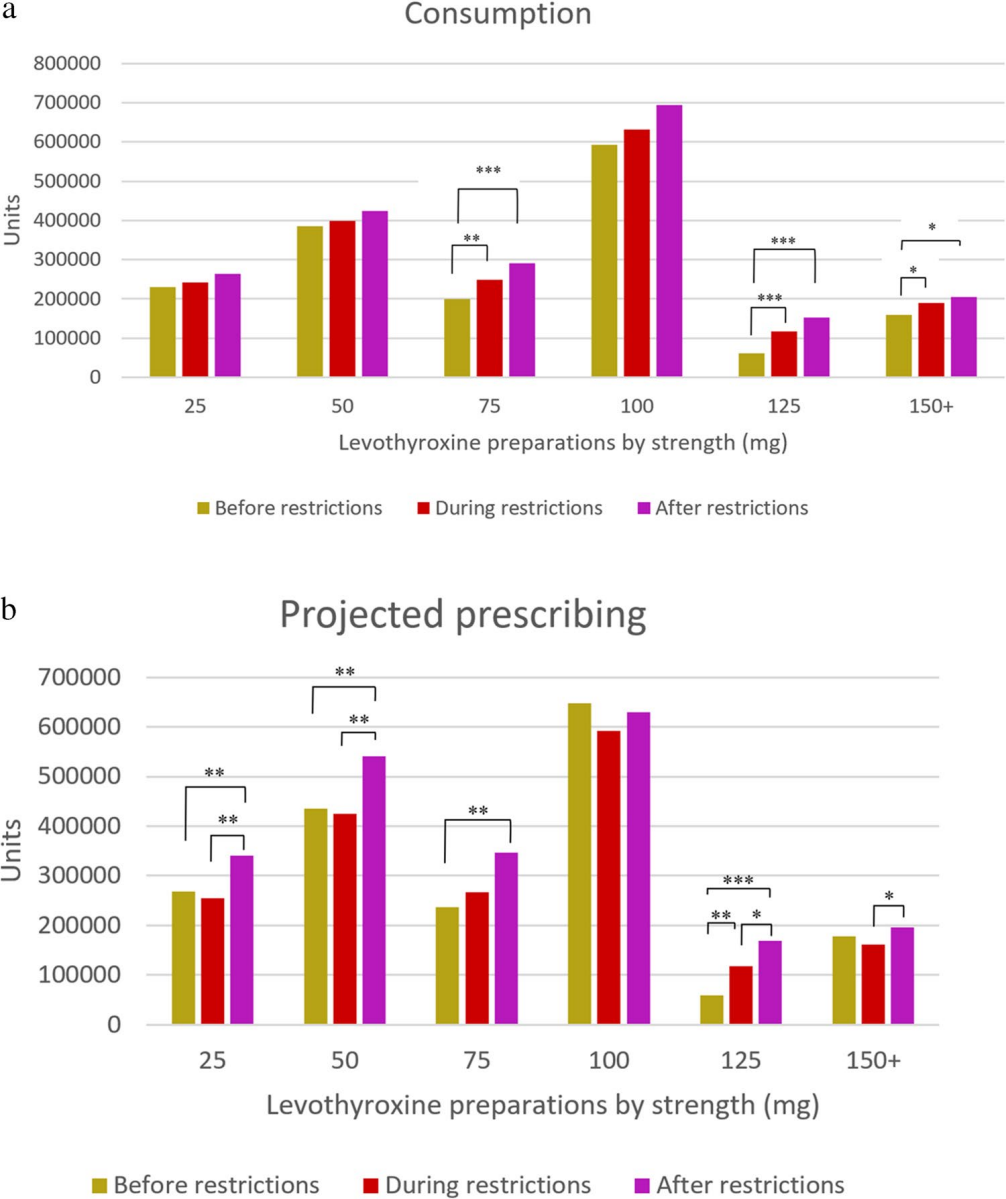

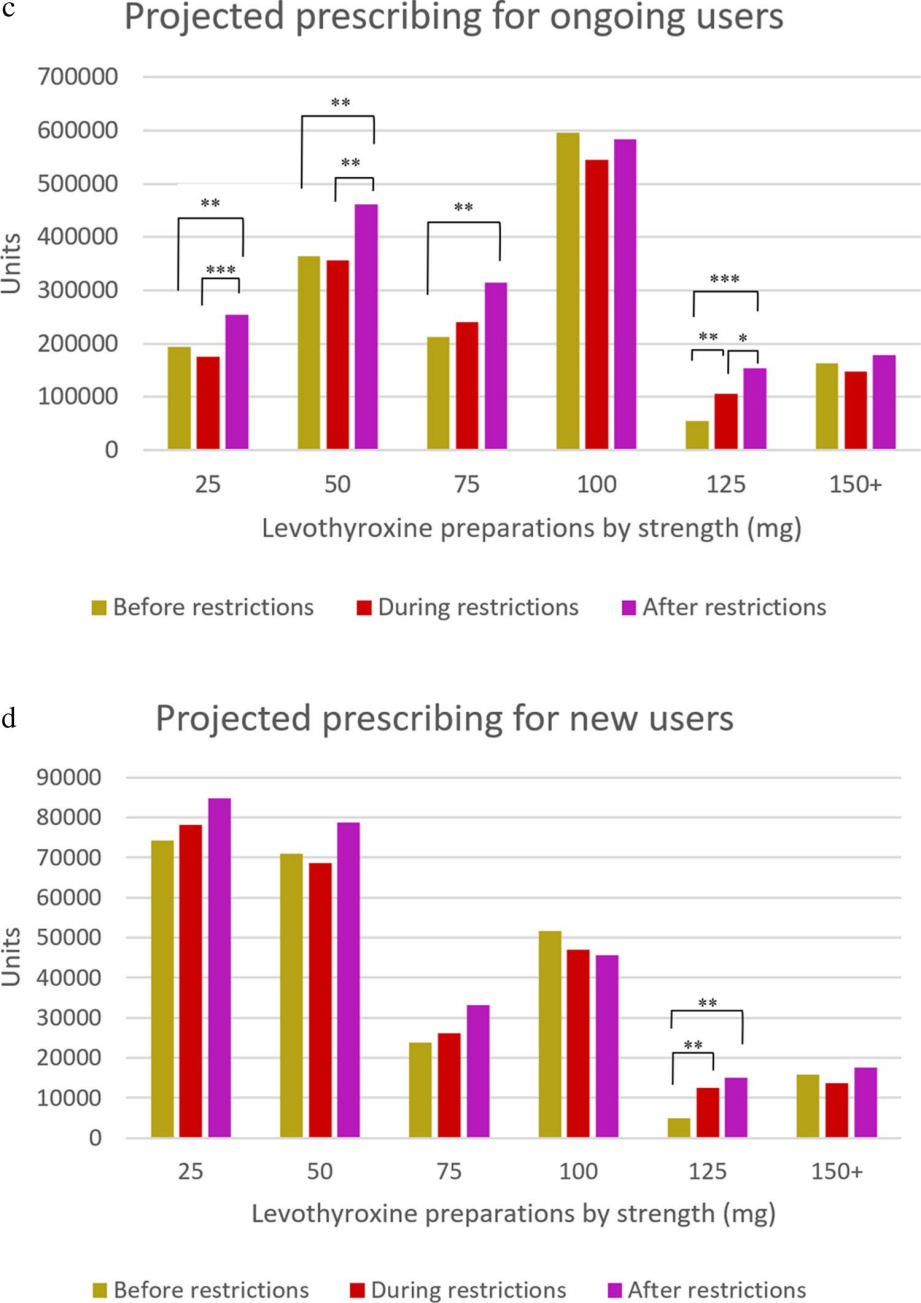
Trends and comparisons of average **a** monthly consumption and quarterly projected prescribing of thyroid hormone preparations for **b** overall users, **c** ongoing users, and **d** new users across COVID-19 restriction–associated periods. ***p < 0.001; **p < 0.01; *p < 0.05

The most commonly consumed levothyroxine doses were 100 mcg (35.2%), 50 mcg (22.4%), and 25 mcg (13.5%), respectively. Strength-based subgroup analyses revealed that consumption of 75-mcg and 125-mcg preparations showed an increasing trend in subsequent periods compared to BfR (*p* < 0.01 for all comparisons, Fig. 3a). The most commonly prescribed doses based on projections were 100 mcg and 50 mcg, both for overall (33.1% and 23.8%, respectively) and for ongoing users (34.4% and 23.2%, respectively). Both overall and ongoing projected levothyroxine prescribing yielded an increasing trend for the 100-mcg preparations across the periods, with higher levels of prescribing for the 25-mcg and 50-mcg preparations observed in AfR compared to earlier periods (*p* < 0.01 for both comparisons). Moreover, projected prescribing of 125-mcg preparations was increased consistently across the periods (*p* < 0.05 for all comparisons, Fig. 3b–c). The most commonly prescribed preparations for new users based on projections were 25 mcg (31.2%), 50 mcg (28.7%), and 100 mcg (19.7%), respectively. Among these, only 125-mcg preparations showed an increasing projected prescribing trend in subsequent periods compared to BfR (*p* < 0.01 for both comparisons, Fig. 3d).

## Discussion

This study, which evaluated the impact of the COVID-19 pandemic and resulting circumstances on hypothyroidism management in Turkey through population-wide drug utilization data, revealed a slight and consistent increase in THP consumption following the onset of the pandemic. In contrast, projected prescribing DID levels of THPs declined during restrictions, except for new users, diverging from consumption trends and implying a potential reduction in healthcare access. Additionally, the post-pandemic increase in the prescribing of these drugs, particularly more pronounced among chronic users, might suggest both normalization of the extraordinary pandemic circumstances and a potentially increased need for pharmacotherapy during this period.

Composed almost entirely of levothyroxine, THP consumption exhibited a moderate upward trend across the subsequent periods, with a one-third increase from BfR to AfR. Moreover, expenditure trends mirrored this rise in consumption. Although pharmacoepidemiologic studies on levothyroxine use remain limited, a multinational drug utilization study reported a similar escalating trend from 2005 to 2011 (Frank et al. 2014). In contrast to consumption and cost trends, THP prescribing levels declined in DuR but rebounded in AfR in terms of DID. This pattern was also observed in those issued for ongoing users, which accounted for almost nine-tenths of all THPs prescribed. A key factor potentially explaining this fluctuation is the reduced access to healthcare services during the pandemic. Indeed, a systematic review of 81 studies from various countries reported an approximately one-third reduction in healthcare utilization in this period, with a more pronounced decline among individuals with less severe illnesses (Moynihan et al. 2021). Furthermore, policies aimed at reducing in-person hospital visits, notably allowing patients to obtain medications directly from pharmacies without renewal of prescription, might have enabled ongoing THP users to obtain their medications directly from pharmacies without consulting a physician (Turkish Medicines and Medical Devices Agency 2020). The sustained levels of consumption during restrictions imply the effect of this intervention. Moreover, the subsequent increase in consumption observed in AfR might be partly linked to the needs of chronic patients, as a study conducted during the pandemic reported that levothyroxine users who experienced delays in follow-up examinations and reduced healthcare contact exhibited greater TSH fluctuations (Inoue et al. 2022). The observed rise in THP prescribing for ongoing users in AfR could further support this interpretation, along with normalization of healthcare access.

The impact of COVID-19 infection on the prevalence of hypothyroidism remains debatable. While some studies reported an increased number of hypothyroidism presentations during the pandemic (Malik et al. 2021; Burekovic et al. 2022), existing evidence suggests that COVID-19 has minimal direct effect on its development (Wei and Zhang 2023). In our population-level data, which is not sufficiently detailed to directly assess such changes, the increase observed from DuR to AfR was rather modest, aligning with the notion of a limited impact, if any.

Levothyroxine dosing varies by age, gender, and weight, typically ranging from 1.2 to 1.6 mcg/kg (approximately 75–100 mcg/day) in patients with partial thyroid reserve, with higher doses generally required for those without thyroid tissue and lower doses are used for subclinical hypothyroidism (Garber et al. 2012; Turkish Endocrinology and Metabolism Society 2023). Given the narrow therapeutic index of the medication, dose adjustments are usually made in 25-mcg increments or reductions (Surana et al. 2017). The most commonly used formulations are 50-mcg and 100-mcg tablets (Jonklaas and DeSale 2020), which align with our findings as those were the most commonly consumed and prescribed doses. These two levothyroxine preparations have their advantages in ease of splitting and suitability for alternate-day dosing. While modest increases were observed in the consumption and prescribing of all strengths, a marked rise in the prescribing of 25-mcg, 50-mcg, and 125-mcg doses, particularly among ongoing users following easing of restrictions, was noteworthy. The increase in these strengths observed in AfR may reflect the overall upward trend, but the variation in changes across different strengths could partly be influenced by dose titration among ongoing users. However, the epidemiological nature of the study and the absence of individual-level clinical data might prevent definitive conclusions regarding the underlying cause. Further research incorporating patient-level clinical information is warranted to validate this interpretation.

THP prescribing for new users followed a stable trend throughout periods, with 25-mcg and 50-mcg levothyroxine tablets being the most commonly prescribed doses. This prescribing pattern may imply that a substantial proportion of these patients may have been treated for subclinical hypothyroidism. A recent meta-analysis conducted across Europe reported that the prevalence of undiagnosed hypothyroidism, predominantly subclinical, reached up to 5% (Mendes et al. 2019). Although pharmacotherapy is generally not required for milder cases of subclinical hypothyroidism, when indicated, levothyroxine is typically initiated at a lower dose than that used for overt hypothyroidism (Garber et al. 2012; Calissendorff and Falhammar 2020).

The results of the study should be interpreted with consideration of its limitations. Although the drug consumption data used in the study cover the entire country, they are derived from the distribution of products from pharmaceutical warehouses to retail pharmacies and do not directly indicate the exact quantities consumed by patients. The study mainly relies on population-level data, which limits detailed patient-specific inferences. Additionally, the absence of direct patient data limited the ability to evaluate differences in THP consumption and prescribing trends by age and gender. The prescribing data were based on nationwide projections; therefore, potential margins of error should be taken into account. The euro/Turkish lira exchange rate determined by the health authority, which is updated annually or every few months, was used in calculating THP costs. This approach may have hindered the accurate reflection of short-term fluctuations in the results. The DDD value for levothyroxine is set at 150 mcg, leading to approximately 90% of levothyroxine usage—comprising the majority of consumed and prescribed THPs—falling below this dose. Both new cases and patients with subclinical hypothyroidism are typically managed with levothyroxine doses significantly lower than the DDD, leaving a very limited number of patients reaching this threshold. Consequently, inferences regarding consumption trends should be interpreted with this context in mind. Furthermore, the lack of thyroid function test data for THP users is a significant limitation, as this parameter is a key determinant in dose adjustment. AfR was limited to nine months due to the February 2023 earthquake, which impacted 11 of Turkey’s 81 provinces. The earthquake, which caused over 50,000 fatalities and widespread, severe property damage, led to the early termination of AfR in December 2022 to prevent potential disruptions in healthcare services and their possible impact on drug consumption from influencing the study results (Manirambona et al. 2023).

## Conclusions

Our study demonstrated that the use of THPs, predominantly levothyroxine, exhibited a modest upward trend following the onset of the COVID-19 pandemic. The divergence in consumption and prescribing trends observed in DuR suggests that patients were able to obtain their medications despite limited access to healthcare services. Additionally, the more pronounced increase in THP use after the easing of pandemic restrictions might be attributed to the extraordinary conditions during the pandemic, along with a potentially increased need for pharmacotherapy. The findings of the study are expected to enhance understanding of the direct and indirect impacts of the pandemic on health care and aid in strategic planning to address these effects, thereby supporting the management of future health crises. The long-term impacts of the pandemic remain unclear, highlighting the need for further comprehensive research and the implementation of precautionary measures in the future.

## Data Availability

The data underlying the findings of this study are available from the corresponding author upon reasonable request.
